# Absence perception and the philosophy of zero

**DOI:** 10.1007/s11229-019-02220-x

**Published:** 2019-05-06

**Authors:** Neil Barton

**Affiliations:** Kurt Gödel Research Center for Mathematical Logic (KGRC), Währinger Straße, 25, 1090 Vienna, Austria

**Keywords:** Number cognition, Zero, Absence perception, Ontogeny

## Abstract

Zero provides a challenge for philosophers of mathematics with realist inclinations. On the one hand it is a bona fide cardinal number, yet on the other it is linked to ideas of nothingness and non-being. This paper provides an analysis of the epistemology and metaphysics of zero. We develop several constraints and then argue that a satisfactory account of zero can be obtained by integrating (1) an account of numbers as properties of collections, (2) work on the philosophy of absences, and (3) recent work in numerical cognition and ontogenetic studies.

## Introduction

Zero is an intriguing number both mathematically and philosophically. Mathematically, a concept of zero plays an important role in our theories of natural, integer, and real numbers. For instance, when considering an algebraic structure (e.g., a group) under addition, zero often serves as the identity element (since for any number *n*, $n+0=n$). Philosophically, our understanding of zero is tied up with classical questions concerning the status of non-being, finding consideration already in the work of Parmenidies, Plato, and Aristotle, through the Continental tradition, and right up to contemporary philosophical debates.

Since there are different specific meanings in mathematics for the term “zero” (for example, in the different number systems), we will examine the epistemology and metaphysics of zero with respect to the *cardinal* number zero, where by (finite^1^) cardinal numbers we mean those numbers that correspond to responses to questions of the form “How many $\Phi$?”, where $\Phi$ is some descriptor of a collection of discrete individuals. We will use the term “collection” in this paper to talk about any collection-like reference to objects, for example we could be referring to singularly to a (semi)set of some objects, plurally to those objects considered together, or to a property/concept extension (and we do not commit to “collections” in this broad sense being extensional). The point of this non-committal stance is simply to make our discussion as general as possible. While we will mostly use the term “zero” to refer to the cardinal number, we shall occasionally use it to refer to the ‘zero’ of other number systems. Where appropriate we will indicate use, however generally we will let context determine meaning.

The core issue concerning zero (that we explain in more detail below) is that it exhibits something of a dual nature. On the one hand, we regard it as a perfectly legitimate cardinal number, on a par with other mathematical entities, and with which we can meaningfully compute. On the other, it represents nothingness: While I can have an experience of two or three objects, an experience of zero objects seems difficult to conceptualise—there would simply be nothing to experience in such a situation. We might then wonder how we should think of zero, both epistemologically and ontologically. One response would be to be an extreme kind of formalist or fictionalist about mathematics, and then let our philosophy of notation and proof-theory or fictions deal with the question of zero. While this is a possibility, in this paper I want to consider how we might account for zero on *Realist* perspectives, since the problem of accounting for zero’s null-like nature then becomes more acute. Therefore we make the following two-part assumption from the get-go:

**Mathematical Realism.**^2^

(i) Mathematics is about a mind-independent realm of mathematical entities (we do not commit to their being bona fide objects^3^) which require an epistemology of how we get to know them.
(ii) A mathematical sentence is true or false (or neither) depending on whether or not the language used and its interpretation correctly describes the world, and this interpretation should match our semantics for natural language as closely as possible^4^ (i.e., *some* sort of correspondence theory of truth is appropriate for mathematics).

The puzzle facing us then is the following:

**Main Question.** How should we understand zero as a mathematical entity? In particular:

(A) How should we conceive of it ontologically?
(B) How is it able to represent nothingness?
(C) How is the cardinal number zero linked to similar uses of the term “0” in various technical scenarios?
(D) How can we provide an adequate epistemology for zero, in particular how we come to know about the cardinal number?

In this paper we argue for the following claims:

1. We can think of zero as a property of *collections* instantiated by empty collections (or *the* empty collection, if one is committed to the extensionality of collections).
2. Cognition of zero can be understood as a species of *absence perception*.
3. An epistemology for zero can be provided by a bootstrapping procedure, and applying considerations from:
  - Numerical cognition.
  - An understanding of counting procedures.
  - Uses of algebraic rules.
  - Our ability to provide descriptions of numbers.

This yields an account of zero on which it can be viewed as fundamentally of the same ontological kind as other numbers, with a similar epistemology based on an integration of cognition of instances of number properties with a broader epistemology of mathematics, yet on which its distinctive null-like features and technical roles are accounted for.

We thus answer (A)–(D) as follows: (A) is answered by point (1) above; zero is a property applying to collections. Our account answers (B) via (2); we hold that cognition of zero can be understood as a species of *absence perception*, and this gives it its distinctive null-like role. Our answer to (C) also relates to point (2); we contend that many of the various uses of the term “0” in mathematics also correspond to absences of particular kinds (hence the commonality in certain properties). (D) is answered by point (3)—we can blend together our epistemology of various areas including descriptions, an understanding of algebraic rules, counting procedures, and accounts of number from the cognitive sciences in providing an epistemology for zero.

Here’s the plan: First (Sect. 1), we’ll argue for some desiderata on a realist account of zero. Specifically we argue that (i) zero should be ontologically similar to the other numbers, (ii) we should have an account of how zero is phenomenologically able to represent nothingness, (iii) our theory of zero should explain how zero-like number concepts can fulfill similar technical roles in different contexts, and (iv) we should provide an epistemology for zero that incorporates these features. Next (Sect. 2), we’ll argue against some otherwise tempting ontological accounts of zero; (i) as a position in a structure and (ii) as identified with the empty set. Each we will argue fails to meet at least one of the desiderata of Sect. 1. We’ll propose instead that zero should be understood as a *property* instantiated by collections. We will then argue (Sect. 3) that an epistemological story for zero can be obtained by integrating considerations regarding (i) logic and descriptions, (ii) the algebraic rules attaching to zero, (iii) counting procedures, and (iv) evidence of cognition of instances of numerical properties from the cognitive sciences. We then (Sect. 4) argue that further understanding of zero can be obtained by considering certain cases of zero cognition as species of absence perception. Next (Sect. 5) we consider some salient objections to the view we proposed, in particular relating to the relatively late historical and ontogenetic development of zero concepts. Finally (Sect. 6) we conclude with some open questions.

## Desiderata on an account

We’ll begin with desiderata on a Realist account of the ontology and epistemology of zero. It is often easiest to see desiderata by considering *clearly unsatisfactory* responses to our main question, and this is the strategy we shall initially adopt here.

One putative solution to the problem posed in the introduction would be to acknowledge that zero is problematic, and accept that we should just be fictionalists or formalists about zero, whilst retaining Realism concerning the other numbers.^5^

Why is this account bad? Well, aside from the fact that it seems rather *ad hoc*, regarding zero as fundamentally *ontologically* different from the other numbers will result in a less theoretically elegant correspondence theory of truth on the realist’s picture. For example, consider the sentence:

“There are exactly six cardinal numbers less than six.”

This is true on the correspondence theory just in case there *really are* six cardinal numbers smaller than six (and presumably there are, since as we have set things up “zero” is the appropriate response to the question “How many scorpions are currently sat on my shoulder right now?”). But, given the current account of zero, this is false: There are really five cardinal numbers smaller than six (the numbers 1–5). We would thus require a version of the correspondence theory on which some way of dealing with fictions in contrast to the other numbers is incorporated, reducing the simplicity of the theory.^6^ If an account could avoid these kinds of awkward changes it would (*ceteris paribus*) be a better theory. We therefore propose the following:

**Ontological Constraint.** Our account of zero should have it ontologically on a par with the other finite cardinal numbers, in that it is of the same metaphysical kind and meshes easily with a correspondence theory of truth (again, we emphasise that in providing a correspondence theory, numbers need not be bona fide *objects*, but could be real entities of some other kind such as properties or Fregean concepts).

As noted in the Introduction, a feature of zero important for its role in our thought is how it interacts with concepts of nothingness and non-being. For instance, if you have zero of a certain kind of object you do not have any such objects at all. This contrasts with other cardinal numbers where there are some objects present whenever you have a collection of objects of the required cardinality. This yields the following constraint:

**The Phenomenological Constraint.** Any philosophy of zero should account for why it represents nothingness within our phenomenological experience.

This phenomenological role for zero in representing nothingness is backed up by its technical use. As remarked earlier, in algebraic structures under addition “0” is used to denote the identity element (since for any number or quantity *x*, $x+0=x$). It also has interesting interactions with multiplication and division. Since “0” represents nothingness, any multiplication of a number by 0 yields 0 (i.e., zero is an absorbing element under multiplication), and 0 has no multiplicative inverse (since, viewing multiplication as repeated addition, any number of times you add nothingness to itself will fail to yield any positive quantity). Moreover, 0 fulfils these roles in a variety of technical contexts: Acting as both the identity element (under addition) and an absorbing element (under multiplication) in each of the integer ring, and rational and real fields. While “0” is simply used to denote the required element of any algebraic structure of the required form, it is also the case that this use of the term “0” was extracted from properties of existing number systems (e.g., the natural, rational, and real numbers) long after they were initially studied; uses of zero-like concepts occur far back in human history^7^ whereas the study of abstract algebra really begins with Euler in the eighteenth century. This yields the following constraint:

**The Technical Constraint.** Explain how the cardinal number zero interacts with use of the term “0” in a variety of technical contexts (for example in the integers and reals, as well as their algebraic counterparts the integer ring and real field).^8^

Immediately there is something of a tension between the three constraints. For, on the one hand, we want our account of zero to put it on an ontological par with the other numbers. However, on the other hand, we require an account that explains why zero fulfils the peculiar phenomenological role that it does and how it is linked to distinctive uses of the term “0” in technical practice, in particular with respect to the fact that it somehow represents nothingness. This suggests that there will be deep issues in providing an epistemology for zero; one might think that the epistemological story should be both similar to that of the other cardinal numbers (since zero is to be of the same ontological kind), yet also in some sense unique (since zero has very distinctive phenomenological and mathematical properties). We therefore add the following constraint:

**The Epistemological Constraint.** Provide a satisfactory epistemological story concerning (a) how we come to obtain a concept of zero, and (b) how we have cognitive access to zero (and hence know facts about zero). Moreover, we should (c) do so in a way that accounts for zero’s similarity to other numbers whilst making it clear how zero is unique.

As we shall see later, we think that there is an account of zero that satisfies these constraints by combining existing work on the philosophy of number cognition, ontogeny, and absences. For now, however, we move on to consideration of the competition.

## The ontology of zero

In this section, we’ll consider various different existing accounts of zero, and explain why they fail to satisfy one or more of the constraints we outlined above. We’ll then provide our positive proposal, and explain how it satisfies the constraints.

### A position in a structure

One suggestion would be to say that zero is a position in a structure. One might hold the following view:

**Structuralism.** Mathematical talk should be understood as fundamentally about *structures*, and mathematical reference should be understood as reference to positions in these structures.^9^

Zero could then be the 0-position in some structure that we then choose to identify as the structure comprising the finite cardinals (such as the structure of natural numbers).

The view of zero as a position in a structure performs well with respect to the *Ontological Constraint*. Zero is to be conceived of as a position in a (relevant kind of) structure, much as the other numbers. Moreover, the Technical Constraint is satisfied since the available attendant isomorphisms on the structuralist picture facilitate the consideration of how zero can play the same role in different structural frameworks. For example, if we are thinking of the cardinal number zero as the 0-position of the natural number structure, we can explain how we might then derive the integer structure by adding additive inverses. Looking at the integer ring instantiated by this structure, we see that the 0-position has the property of an identity element under addition. Moreover, there will be an isomorphism between the structure of the integer ring and a substructure of the real field, on which 0 in the integer ring will be mapped onto 0 in the field structure, and an isomorphism between the natural numbers and a substructure of the integers (i.e., the positive integers starting from 0). We thus have a detailed account (which can be relativised to the various accounts of Structuralism available) of how the cardinal number zero, conceived of as a position is some structure, interacts with the other structures of mathematics (such as in abstract algebra).

However, such a picture fails to account for how zero satisfies the Phenomenological Constraint. 0-positions in structures fail to have any non-being-like qualities; they differ only in their relational properties and are intrinsically the same as the other positions in structures. The initial position in the natural number structure, for instance, is just an initial position, but does not represent nothingness in any way apart from its structural properties. Of course, these structural properties give it some features that allow it to do the mathematical job we desire from zero in representing nothingness, but the position *itself* does not have these qualities. We see this as partial violation of the Phenomenological Constraint. In this sense, the kind of realism we have adopted in this paper, as seen through the lens of the four constraints, is unabashedly non-structuralist—for this kind of realist there is more to numbers (especially zero), than mere structural relationships. Later, when we provide our positive proposal, we will argue that we can account for the structural properties of zero whilst at the same time linking it to ideas of nothingness. For now though, we reject the view of zero as a position in a structure as violating the Phenomenological Constraint (given our initial set up).^10^

### The empty set

A different option would be to identify zero with the empty set, and rely on the representative qualities of set theory and an attendant epistemology of sets to do the work. We might, for example, hold the following strong view.

**Strong Set-Theoretic Reductionism.** Every mathematical object is a set.

Then we could simply identify zero with the empty set, after all, in nearly all theories of the ordinals (including our canonical one: the von Neumann ordinals) the empty set is the representative for 0. We would then easily satisfy the Ontological Constraint (the empty set is, after all, a *set* just like the other numbers), and we would, prima facie, satisfy the Phenomenological Constraint (since the empty set is, after all, *empty*) *and* set theory provides a clear mathematical picture of how it plays the role it does.

Aside from the fact that we might dispute such a strong set-theoretic reductionism^11^, it is when we examine the Epistemological Constraint that this account begins to appear problematic. The simple difficulty is that by reducing zero to the empty set, we have substituted one problem of non-being with another—it seems equally tricky to explain how we get to know the empty set as it does the number zero.

Not many accounts of the empty set exist in the literature. One can be extracted from Maddy (1990b) and Maddy (1990a), which suggest (for the physicallistically-inclined philosopher) that since we just require that the empty set be able to play a certain technical role, we can just pick any old object we like to its duty.^12^ One choice here (though not exactly the one taken by Maddy) is to build the hierarchy from a single urelement; since an urelement has no members, it will have all the regular mathematical properties we require of the empty set. Moreover, since the Maddy (1998) holds that sets are *perceivable* and *physically located*, there is *literally* a set-theoretic hierarchy located wherever objects are. The epistemology of the empty set then reduces to the (non-mysterious) epistemology of the relevant object we pick.

One key problem with this account is that it makes the choice of the empty set (and indeed derivatively all pure sets) seem arbitrary and open to modal variation. Might we pick a contingently existing object? If so it seems like the necessity of mathematical truth becomes dubious, since the empty set (and hence all the relevant pure sets) may *fail* to exist at certain possible worlds. Indeed, if we pick a destructible object, it may fail to exist at *this* world at some point in the future.

This problem is partially assuaged by Lewis (1991)’s account of the empty set, somewhat similar to Maddy’s in spirit (in that he picks a particular object), and on which he avoids these problems by being a staunch four-dimensionalist about time, a strong modal realist, and then identifying the empty set with the mereological fusion of absolutely everything. However, we might worry about making our account of the empty set dependent upon such strong metaphysical assumptions concerning the nature of temporal and modal space.

A different, less ontologically-loaded, response on behalf of the Maddy-style theorist is to argue that they were never trying to point to *the* identity of the empty set, but rather set theory could be viewed as explaining what *would* be true on any hierarchy of the correct kind, and claims about the empty set should be understood in this vein. Then worries of contingent existence disappear; we remain neutral on any *one* urelement being the empty set.

This response goes so far, but unfortunately does not pass muster. The key problem is that we would then have a slew of difficult philosophical questions to answer about the nature of our talk concerning the empty set, some of which are problematic from the perspective of the Ontological Constraint. For example, should we now say that there are many empty sets or just one? The former is *strictly speaking* correct, whereas the latter is what would be true given the relativised interpretation of our mathematical talk. Unfortunately in such a case we have to modify the correspondence theory *slightly*, since we cannot take our talk at face-value (it has to be appropriately relativised). A philosophy of zero that is able to avoid these sorts of deformations in the correspondence theory is thus (in this respect) preferable.

More seriously for the Ontological Constraint, since the empty set is now not (strictly speaking) a *set*, whereas the other sets *are* sets, we have to do additional violence (even within a particular relativisation) to the correspondence theory of truth. In particular, the empty set is not of the same fundamental kind as other mathematical objects (which are, on Maddy’s view, bona fide *sets*), and so we require a work-around in the version of the correspondence theory we employ.

### A size property of collections

None of the accounts we have considered so far are able to fully meet the constraints of Sect. 1. However, useful observations emerged from our discussion. The structuralist perspective was able to easily account for various algebraic properties attaching to zero. And one of our responses to the proposal that zero could be identified as the empty set (namely that it seemed to simply push our epistemological difficulties back one stage), showed that there will be links between the epistemology of the empty set and that of zero (even if we are not identifying zero with the empty set), specifically in that zero is the cardinality of the empty set. In this subsection, we’ll *very briefly* outline the bones of our proposal, before filling out an epistemological story in in the next section.

One response to the ontology of cardinal numbers has recently been defended by Giaquinto (2017), and argues that cardinal numbers are size properties of collections. Giaquinto states his view as follows:

“...cardinal numbers are sizes of sets. Set size is a discrete magnitude; in other respects, it is much like length, duration and weight (which we tend to think of as dense and continuous magnitudes). The set-size view takes our pretheoretical thought and talk literally: ‘class size’ in normal parlance refers to the number of pupils in a class, and ‘family size’ refers to the number of family members.” (Giaquinto 2017, p. 2)

Since we are couching our discourse in terms of collections rather than sets, we shall refer to the view that cardinal numbers are sizes of collections of discrete objects as the **Collection Size Hypothesis**. From the off, it seems that the Collection Size Hypothesis combines well with Realism as we have outlined it. Each numeral denotes a single cardinal number and numbers can be mind-independent entities (as long as properties are). Moreover, the Collection Size Hypothesis seems at least consistent with the view that there are infinitely many (finite) cardinal numbers.^13^ While there is perhaps the question of whether there are infinitely-many such properties (as it seems cardinal arithmetic requires) if the universe is finite, as long as one accepts that properties can exist uninstantiated by the (physical) world, one can obtain the required properties. This is a hypothesis that seems to mesh well with Realism, even if it is not implied by it. It seems then that the Collection Size Hypothesis is at least a plausible option for our account of cardinal numbers. Zero under this proposal is then the cardinality property applying to (the) empty collection(s). In fact, we will argue for the following claims:

1. Zero is a *size property* of collections, instantiated by empty collections (or *the* empty collection, if one is committed to the extensionality of collections).
2. Cognition of zero can also be understood (in certain cases) via *absence perception*.

We will argue for claim (2.) in more detail in Sect. 4. For now, we wish to point out that the account is promising, at least regarding the four constraints. The Ontological Constraint is very easily satisfied. Zero is understood as a property corresponding to collection-size just like the other natural numbers. Thus conceived of as a property, it is in exactly the same ontological standing. As a result, we can provide a correspondence-style theory of truth without having to treat zero especially differently from the other finite cardinals.

Moreover, the Phenomenological Constraint is also clearly satisfied. Though we have acknowledged that zero is a collection-size property, it is the *only* cardinal number where the absence of objects of the relevant kind is experienced in a token experience of an instance of zero. We will argue for this absence claim in detail later, however *given* that we should understand zero this way, we would have an explanation of why it represents nothingness; cognition of empty collections results in a representation of absence in experience.

Regarding the Technical Constraint, we contend that our account provides the foundation for a generalisation to various contexts, including the set theory, and the integer, real, and rational numbers (and their associated algebraic structures). Concerning set theory, if we think of the empty set as a particular kind of collection, then zero is precisely the cardinality of the empty set.^14^ If we think of the natural numbers as corresponding to the cardinal numbers, and then the integers obtained from the natural numbers by adding additive inverses, then the zero-position of the integers and zero are both kinds of absence; the zero of the integers is the only integer an absence of magnitude. Thinking of the real numbers as giving mathematical structure to ratios of lengths, we can think of the origin as providing the point from which the length of particular lines can be measured. The real number 0 can then be thought of as an *absence* of positive length (since the distance from the point 0 to the point 0 is zero). This explains the relationship between the cardinal number zero and the real number zero; both can characterised by absences of a sort, the former in numerosity and the latter in length. These examples then show why the zero-positions in the algebraic structures (e.g., integer ring, real field) abstracted from these number systems have the properties they do. The algebraic rules for addition and multiplication are not simply defined out-of-the-blue, but relate to the deeper philosophical issue of how arithmetical operations and absences interact. When an absence is added to a number, you can never generate a number of a greater magnitude (i.e., zero-positions are identity elements under addition). Moreover, no matter how many times you repeat an absence, you will never generate any positive magnitude, nor can you generate positive magnitude if you take an absence of instances of a particular number (i.e., zero-positions are absorbing elements under multiplication).

Our answer to the Technical Constraint is somewhat partial in that a full account of how zero figures in many mathematical contexts would require a satisfactory epistemology for each of these contexts, each of which would require lengthy treatments in themselves. One might, for example, not take the view that real numbers are to be understood as ratios of lengths. However, our position is *flexible* in that it can be integrated into a wide variety of different accounts, and as long as they admit of a notion of absence, it performs well in explaining the role of different zero positions.

As it stands though, we have not explained the distinctive epistemology for zero, and thus a response to the Epistemological Constraint remains wanting. It is to this problem that we now turn.

## The epistemology of zero

There are two main kinds of problem in providing an epistemology for our account of zero that meets the Epistemological Constraint. The first is the general problem of providing a satisfactory epistemology for the Collection Size Hypothesis. The second is to explain how zero specifically could fit into such an account. We tackle these problems in turn, before arguing that cognition of zero should be understood as related to absence perception.

### Bootstrapping the cardinal numbers

A key problem (especially in light of Benacerraf 1973) in providing an epistemology for the Collection Size Hypothesis is to explain how we can have cognitive access to these properties. After all, these properties are meant to be abstract, non-spatiotemporal, and acausal. So, how can we know facts about them? Giaquinto (2017) addresses this “cognitive access problem” by pointing out that in order to have cognitive access to a property, it is enough to be able have cognitive access to some of its instances, and access to instances is often manifested in recognise-and-distinguish abilities. For example, I can recognise an instance of letter type (where a letter type is an abstract visual form) and distinguish it from other letter types. Similarly, I can recognise an instance of the riff (where a riff is an abstract aural form) from Led Zeppelin’s ‘Black Dog’ and distinguish it from that of ‘Whole Lotta Love’.

As Giaquinto (2017) points out:

“Such a recognize-and-distinguish ability requires one to have an enduring representation of the sensory form. Recognizing something as an instance of the form requires an interaction between (i) a representation produced by current perceptual input and (ii) an enduring representation of the form. We may think of the interaction loosely as a comparison process which, in the case of recognition, has a positive outcome. Distinguishing between instances and non-instances involves the ability, when presented with a non-instance of the form, to perceive that it is not an instance. For this, a necessary condition will be that the ‘comparison’ process between the representation produced by current perceptual input and the enduring representation of the form has a negative outcome.” (Giaquinto 2017, p. 3)

One way then to provide a satisfactory epistemology for the view that cardinal numbers are properties of collections (particularly with respect to the cognitive access problem), is to provide an account of how we obtain a concept that facilitates such recognise-and-distinguish abilities. We can come to such an account via “bootstrapping”; explaining how elements of “core cognition” (i.e., “highly structured innate mechanisms designed to build representations with specific content”^15^) are combined to yield a concept with more representational power than the parts on their own.

Such a bootstrapping procedure is suggested in Carey (2009). Work by several scholars indicates that there seem to be at least two systems of “core cognition” capable of representing numerical properties.^16^ The first, known as “subitizing” allows us to recognise and distinguish small numbers without counting. For example, Benoit et al. (2004) examined a sample of forty-eight 3–6 year olds, and found that they were, given a presentation of 1–6 dots for 800 ms (i.e., too quickly to count), able to recognise and distinguish presentations of under 3 dots, and younger members of the sample (i.e., approximately three years old) performed better when dealing with short simultaneous presentations rather than sequential presentations that would allow them to count. Subitizing, however, appears to not function with collections greater than four. The second is the known as the “approximate number system” or “ANS”, and allows us to approximately discriminate larger cardinalities. For example Barth et al. (2003) showed that adults are capable of comparing arrays of between 10, 20, or 30 dots when flashed in too short a timeframe for counting. This latter system exhibits the so-called “distance effect”; two collections closer in cardinality are harder to discriminate into the larger and the smaller than two collections further apart in cardinality. There is also a ‘size’ phenomenon; it is harder to discriminate a pair of large numbers differing by size *n* than it is a pair of smaller numbers differing by *n*. These abilities are both not specific to humans (having been observed even in rats), and are multi-modal (it does not matter, for example, whether or not the array is visual or aural), and experiments have been conducted to control for various confounding factors (e.g., total surface area of dots in a visual array). Moreover, through fMRI imaging with human subjects and experiments on rhesus monkeys, this perception of numerosities is correlated neurally with increased activation of the parietal cortex.^17^ It appears that numerosities are cognitively encoded on a numerical continuum, with the numbers 1–4 precisely discriminable, and higher numerosities approximately so.^18^

Carey (2009) suggests that this, combined with observations concerning ontogeny, provides a way of acquiring a concept of cardinal number (conceived of as natural numbers). We outline (very briefly and omitting many details) her proposal here. In order to have a representation of cardinal number, as represented by the list of numerals, we need the following:

1. A concept of a (possibly meaningless) list of numerals.
2. An understanding that these numerals represent the cardinality of collections.

Carey (2009)’s proposal is (very) roughly as follows. It is part of core cognition that infants can learn meaningless ordered lists (e.g., “eeny, meeny, meiny, moe”), and they begin by learning the first few numerals in this way. To begin with, they associate “one” with singular reference, and all higher numerals with plural reference to collections larger than one. This is confirmed by studies in which English-learning young (approximately 30 month old) children will hand over one fish when asked for “one fish” but will hand over a plurality of size greater than one when any other number of fish is requested.^19^ This occurs even when such infants possess subitizing cognition and the ANS. Eventually, children learn to associate the numbers 1–4 to collections of the appropriate size, whose cardinalities are represented via subitizing in their experience. They can then induce that the successive numerals are obtained by the operation of adding one. Possession of a notation system on which there is a way of obtaining numerals via successors facilitates an understanding that a representative numeral can be obtained for the precise sizes to which their detection of approximate magnitudes are to be associated. This is again supported by the empirical data, since counting competency is correlated with the ability to associate magnitudes with numerals. For example Lipton and Spelke (2005) analysed the counting competency of a sample of children by asking them to count from certain numbers (e.g., 76, 77, 78,...), before asking them to estimate the number of dots in a visual array presented too quickly to count. They found that skilled counters showed a linear relation between number words and nonsymbolic visual arrays. Unskilled counters on the other hand showed the same linear relation for smaller numbers to which they could count, but not for words corresponding to larger numerosities. Thus, we can explain how elements of core cognition (e.g., subitizing, the ANS, and the learning of inference rules regarding successor) are combined by agents in coming to have a concept of cardinal number.

How might we feed this bootstrapping account into recognise-and-distinguish abilities? Giaquinto (2017) suggests that we can have cognitive access to larger numbers by providing identifying descriptions. Once we have an understanding of how successor is correlated with increase in collection size, we can then understand addition as the repeated taking of successors, multiplication as repeated addition, and exponentiation as repeated multiplication, and superexponentiation as repeated exponentiation.^20^ These allow us to describe progressively more and more numbers using an identifying description. In this way, we are able to explain how the concept of cardinal number obtained by bootstrapping is able to figure into recognise-and-distinguish capabilities, in turn explaining how we have cognitive access to instances of cardinal numbers, dissolving the cognitive access problem and grounding our knowledge of cardinal numbers.

We have skated over many of the details here, and further work in philosophy, the cognitive sciences, and ontogeny will help to fill in the picture, as well as helping to respond to or clarify possible objections. We will not embark on the project of providing a fully complete account of acquisition and employment of our concept of cardinal number here; the core point for our purposes is that we *can* generate bootstrapping accounts to explain how we obtain a concept of cardinal number and use it to provide an epistemology for numbers conceived of as properties of collections. Our challenge is to show how we can generate such an account that incorporates knowledge of zero.

The tasks before us are thus the following:

1. Show that zero can be incorporated into such a bootstrapping account.
2. Argue that in such an account, cognition of zero can be regarded as a species of absence perception.

### Bootstrapping and zero

Consider then the following (somewhat silly) example. Suppose that you are really hungry, and I tell you that there are either three or four sandwiches in the hamper next to me, and you are welcome to eat all of them (suppose I can’t remember whether I packed the last sandwich I made). You are *really* hungry, and it *really* matters to you whether or not there are three or four sandwiches there. You are thus primed to engage your ability to perceive small cardinalities. You open the hamper to find...no sandwiches! (I secretly ate them all on the bus and then lied.) Might we argue that in this case that you experience an instantiation of zero-cardinality of the collection in question? This would then yield the beginnings of an epistemology of zero; instances of zero can figure as part of core cognition (and subsequently fed in to bootstrapping procedures).

While it was long thought that this was not possible,^21^ recent studies on monkeys and children (of 4 years) suggest that such cognition occurs. For example rhesus monkeys are able to discriminate empty sets in numerical selection tasks. Merritt et al. (2009) performed experiments in which rhesus macaques were first trained in numerical matching and ordering tasks (of either 1, 2, 3, 4, 6, 8, or 12 dots), and then their response to possible empty collections examined (in this case, arrays of zero dots). In one experiment, a pair of macaques (Mikulski and Schroeder) were required to match arrays given an initial stimulus, followed by two options for selecting a stimulus of equal cardinality. In a second experiment, Mikulski and a new macaque that had not participated in the first experiment (Feinstein) were required to select the smaller of two stimuli. Similar distance effects were observed in both experiments for tasks involving arrays of cardinality zero. When matching, accuracy was improved when the ‘wrong’ option was further away from 0, and when selecting the smaller numerosity, accuracy improved when the stimulus was further away from zero (so, for example, monkeys found it easier to compare 0 and 6 rather than 0 and 1).^22^ Similar neuronal activation of the parietal cortex as with ordinary numerosity cognition has also been observed in animals when perceiving collections of cardinality zero.^23^ This meshes well with the claim that the distance effect is explained by overlapping neuronal activation that decreases as the numerosity of the stimulus increases (Ramirez-Cardenas et al. 2016; Okuyama et al. 2015; Merritt et al. 2009).^24^

These distance effects have been observed to transfer to 4 year old children on numerosity-matching tasks (Merritt and Brannon 2013). When asked to select the smaller of two visual arrays of cardinality (0, 1, 2, 4, 8) children exhibited distance effects as long as they showed competence in ordering countable symbolic numerals (otherwise they responded at chance). Thus, it seems plausible that in fact numerosity zero is encoded in a similar way to the other numbers. Is such an element of core cognition sufficient to yield a mature conception of zero? The data suggests not. For Merritt and Brannon (2013) showed that while distance effects were observed in children capable of ordering numerals $n \ge 1$, many still failed to order the symbolic number zero correctly. This indicates that numerosity zero can be encoded as part of core cognition without yielding a mature concept of zero on which zero is integrated with the other numbers.

However, these observations can be integrated with other components of learned and core cognition. It has already been mentioned above that the ability to associate numerals and magnitudes as represented by the ANS correlates with counting competence.^25^ Given then the realisation that the successor function has a inverse (i.e., that one can also subtract 1 as well as add 1 to yield the predecessor instead of the successor^26^) we might then consider what the predecessor of 1 might be, and realise that it must be zero (since $1-1 = 0$). Thus, we can use our epistemology of counting practices in bootstrapping zero; it is the number that would have to precede 1.

This idea has been studied in the context of the literature on counting practices in the ontogenetic development of number-concepts in children. Wellman and Miller (1986), for example, used backwards counting songs ending in zero to study children’s competence with zero. In particular, pre-schoolers (namely fifty-seven 3–7 year olds) were tested by being told to count four cubes in front of them out loud, and then count backwards as each cube was taken away (culminating in the final cube being removed). Their results showed that there was a clear association for children to assert that the last cube to be removed was to be associated with “none” or “nothing” (54 out of 57 children), with children later associating this with a number zero that is to be regarded as the smallest number.

Again, we can ask whether or not a ability to manifest a counting procedure sufficient to produce a concept of zero. Again, the data suggests not. Whilst the children in Wellman and Miller (1986) *did* associate “nothing” or “none” with removal of the final cube, it is not clear that they associated this with *zero* as understood as part of a mature number theory. This can be seen from the details of Wellman and Miller (1986) which lend credence to the claim that in the ontogenetic development of zero, a conception of certain features of zero *predevelops* its full integration into counting practices. The original experiment with 57 children contained some additional tasks, in addition to the backwards counting task described earlier (i). (ii) Children were also asked to compare two collections of cardinality three and four, and then to name the “very smallest number”. If the child responded with a number $n>0$, the cube task was repeated with *n* cubes and the child asked again to name the “very smallest number”. (iii) A deck of 11 cards inscribed with the numerals 0–10 was shuffled and the child asked to name each number as the cards were shown. (iv) Fifteen cards containing two different numerals were shown, ten containing pairs from 1–5, and five with “0” paired with a numeral from 1–5. Children were asked to point to the smaller (or larger) of the pair. On tasks (iii) and (iv), children would repeatedly say that 1 was *smaller* than *zero*, even when they were able to perform the backwards counting task and name zero as a numeral corresponding to nothing.^27^ It seems then, if we accept the experimental set-up of Wellman and Miller (1986), then a *full* integration of zero into our conception of number (specifically understanding of 0 as *less than* 1) postdates an understanding of a number associated with the numeral “0” and linked to nothingness, at least as far as ontogenetic development is concerned. This suggests that counting practices alone are not sufficient to deliver a mature conception of zero, even if it does not outright imply it.

Wellman and Miller (1986) identify three key phases of acquisition of a zero concept using tasks (i)–(iv). In phase one, children acquire an ability to name the written numeral. In phase two they are able to associate this numeral with nothing. In phase three, they are able to integrate zero into relationships with other numbers. It is interesting that shortly after zero has been integrated with other cardinal numbers children show a surprising capacity for competency with algebraic rules (such as $a+0 = a$, for any *a*). Wellman and Miller (1986) conducted an experiment on a second group of 48 children (sixteen 5–6 year olds, sixteen 6–7 year olds, and sixteen 8–10 year olds) with a mind to assessing children’s competence in this regard. (v) They were shown cards with the numerals 0–3, and asked questions (given one displayed card with a numeral *n* and one card behind a door), designed to probe comparison and understanding of subtraction and addition rules. For example, a comparison question (designed to probe whether the other numbers were bigger than zero), might be “Someone told me that no matter what is behind the closed door, it will be bigger than *n*. Is that so?” and an addition question might be “Someone told me that no matter what number *m* is behind the closed door, it will be the answer to $m+n$. Is that so?”.^28^ The details of experimental set up and results are available in Wellman and Miller (1986), but to summarise (a) young elementary schoolchildren showed a fair level of competence with the employment of algebraic reasoning involving zero, and (b) they performed better when employing rules concerning zero as opposed to other positive numbers. Wellman and Miller (1986) speculated that children are resorting to rule-based reasoning in virtue of the difficulty of reasoning with zero, but we needn’t commit to this latter claim.

We may interpret the data via a bootstrapping argument as follows. We begin separately with numerical cognition of collections of size zero, a concept of a numeral “0” as part of a meaningless list, and a general concept of nothingness. We then are able to understand that the numeral is linked to nothingness, before linking this to our experiences of numerical cognition of collections of size zero, and subsequently integrating it as a magnitude smaller than the other numbers. We are then able to learn that such a number (since it represents nothing) is easily integrated with certain algebraic rules of reasoning, yielding a mature concept of zero as part of the structure of cardinal numbers. We thus obtain cognitive access to zero via a variety of means. On the one hand, after we possess a mature concept of zero, instances of zero are represented as magnitudes in our cognition. But we are also able to identify zero as the unique predecessor of 1, and as the unique number in the cardinal number structure obeying certain algebraic rules. We thus have a response to the Epistemological Constraint, having explained how we obtain and employ a concept of the cardinal number zero.^29^

## Zero and absence perception

Our responses to the Phenomenological Constraint (and our related responses to the Technical Constraint) depended upon arguing that cognition of instances of zero are linked to absence perception. In this section we suggest that the epistemological story we have provided supports this hypothesis. We have seen that the cognitive science literature lends credence to the claim that instances of zero are represented as part of the cardinal number structure in our experience. The question remains, however: How should we understand the *philosophical* character of the experience correlated with these neurobiological states? This is not a neurobiological question but rather a philosophical one; we need to to explain how these sorts of experience are integrated into a wider theory of perception and cognition.

As it stands, the neurobiological data does not explain in and of itself why zero has such a distinctive character for us. We have an incomplete *philosophical* story; why should zero have the phenomenological and technical character it does, given that it seems that neuronal activation is similar to the other numbers? Certainly, the neuronal activation is literally-speaking different, but what philosophical correlates are there of how experiences of the distinction between tokens of zero and one are different from experiences of the distinction between tokens of two and four? How does our bootstrapping argument integrate with wider philosophical issues concerning recognise-and-distinguish abilities in perception and cognition?

In many of the cases of cognition of zero considered in this paper, we have a case where a subject has been primed to have a cardinal number perception, but where an *absence* is a possible stimulus. Consider, for example, Wellman and Miller (1986)’s backward counting songs where the final cube is removed, or the presentation of visual arrays to children and monkeys in Merritt and Brannon (2013) and Merritt et al. (2009) where a visual array with no dots is presented. In this respect, our somewhat silly sandwich example from earlier turned out to be roughly analogous to many of the *actual* experimental set-ups, and is not so silly after all. We thus make the following claim: In many cases of cognition of instances of zero, we should understand the experience not just as cognition of *number* but also perception of an *absence*.

Certainly, the question of whether absences can be perceived at all is a tricky philosophical question, since on many theories of perception we only perceive present objects or scenes.^30^ This, however, has recently been challenged in the literature on absence perception, for example in the work of Roy Sorensen^31^ and Anna Farennikova.^32^ It would take us too far afield to give a full defence of absence perception, however we can lend plausibility to the epistemological and metaphysical story we are providing by clarifying the exact nature of the absence perception we are proposing.

Sorensen (2008) provides a book-length treatment of absence perception. Many absences that Sorensen considers have determinate sensible qualities (such as holes, gaps, the cold, and shadows). However, other absences do not have this quality. *Silence* is a good example here. Thinking of silence as a relative^33^*absence* of sound, there is no concrete positive *sensation* accompanying a token experience of silence, as there is with seeing darkness or feeling cold. For this reason, Sorensen claims:

“Hearing silence is the most negative of perceptions: there is nothing positive being sensed and no positive sensation representing that absence.” (Sorensen 2008, p. 272)

It is interesting that zero shares many properties with silence. For example, instances of zero cardinality (like silence) can be located; I can both hear the silence *in the cockpit* of an unconscious pilot who has left their microphone on^34^ and have an experience of zero sandwiches *in the hamper*. Moreover, instances of zero are (like silence) both detectable and directly perceivable; if I install a device to the outside of a box that emits a high-pitched tone when the contents of a box contain no items of a certain kind (respectively, when the volume inside the box is below a certain level), I can detect an instance of zero (respectively silence) inside the box without perceiving it. Especially interesting, however, is the following similarity between zero and silence; token perceptions of zero are characterised by a lack of expected stimulation, and so there are no *particular* qualities associated with zero other than its zero-ness (for example, zero is not coloured in the same way as darkness, which is black in colour). This resonates with Sorensen’s account of silence expressed in the quotation above.

Drawing on this character of zero, one can then view perceptions of zero as the kind of absence considered by Farennikova (2013). There she considers a model of absence perception on which absences are understood through *mismatches*. For example, suppose I am looking for and failing to find my keys in the usual expected places. On her picture, I develop a rough visual template of my keys in my working memory and attempt to project it onto my visual surroundings. When the template fails to project accurately onto my surroundings, I am aware of a mismatch between an expectation arising from the working memory projection and the world, yielding a sensation of absence.

Farennikova’s Mismatch Model can be adapted to the current case to provide an elegant interpretation of perception of instances of zero. In a situation of numerosity perception we are projecting possible numbers of objects to be (possibly approximately) matched. In perception of a token occurrence of zero, we have an expectation arising from projection of positive numerosity in a cardinal context mismatched with a lack of any number of things. This is part of what it is for us to *recognise* an instantiation of zero and *distinguish* it from instantiations of the positive finite cardinals. Thus, in addition to understanding zero as a property which can be experienced via *number* cognition we can also understand with via perception of *absence*.

This suggestion that we should understand experience of zero-tokens through an absence perception arising from mismatches might be argued to mesh well with the fact that number cognition is often explained via the use of an *object-tracking* mechanisms.^35^ Thus, an absence of positive numerosity (i.e., a token perception of zero numerosity) may indicate a mismatch between an *object* tracking mechanism and a *lack* of objects. This is rather speculative at this stage, and more evidence for this claim could be obtained by empirical studies, but there is at least the possibility of integrating the different accounts more fully.

So, to sum up the positive account, we have proposed that zero should be understood as follows:

1. A *size* property instantiated by collections.
2. A concept of zero can be obtained via a *bootstrapping* argument, solving the cognitive access problem.
3. Cognition of zero as a magnitude can be understood through a model of *absence* perception as a *mismatch* between projections and the world.

## Objections

We seem to now have an attractive epistemological and metaphysical account of zero for the Realist, responding well to the constraints and indicating ways in which the account can be worked into further study. There are, however, some salient objections. This section explains and addresses these objections, and shows how they help to clarify the account.

*One can expect instances of zero.* In the account we provided above, we argued that we can perceive tokens of zero via *expectation* violation (in the sense of mismatches between the world and expectations arising from projections). However, one might argue that we can in fact *expect* a perception of a token of zero in a situation where we have an absence, thereby challenging the model provided. Suppose I tell a younger sibling to always leave at least one sweet in the jar (out of politeness), but lacking self-control they usually eat all of them. I come down in the morning, expecting to find the jar containing zero sweets, and predictably it does. How can I have a perception of zero numerosity via mismatch if I actually expect zero sweets?

The answer is already dealt with in Farennikova (2013) and we adapt that response to the current context. Despite the fact that I *doxastically* expect there to be no sweets, I nonetheless *project* the positive numerosity of sweets onto my surroundings in an attempt to discern the mismatch and see the absence. In other words, I assess my *doxastic* expectation that there won’t be any sweets by comparing the world against a *perceptual* expectation of seeing a positive numerosity of sweets. Compare the situation in which, against all probability, there is a sweet in the jar. Here, my doxastic expectation is violated, whilst the perceptual expectation I generate through perception is met. It is this latter sense of expectation we have in mind. The objection that I can predict and expect absence in a cardinal context thus conflates two notions of expectation; the expectation that arises from beliefs and the cognitive expectation that arises from projecting in the process of a search.

*Perception of number is multi-modal.* A salient feature of number cognition as magnitudes is that it is independent of a particular modality. For example, in addition to the visual abilities mentioned above, studies on rats have shown that similar systems are in play when the stimulus is aural.^36^ This contrasts with perceptions such as those of colours, which are only instantiated visually.^37^ Consider a case where we are asked to perceive the total number of tones heard and circles displayed on a screen. No circles are displayed and no tones sound. It seems reasonable to suggest, if we accept the current proposal, that this is a *multi-modal* perception of an absence of both visual and auditory stimulus *at the same time*. It is, however, controversial whether or not there are perceptual faculties that are multi-modal, or whether rather distinct perceptual faculties are tied to specific modalities and integrated in cognition. If we reject the possibility of multi-modal perceptual faculties, we would thus have to reject the account provided.

We can respond as follows: If we analyse what we required out of our account of zero above in satisfying the constraints, it was that it (1) could be combined into a bootstrapping account appealing to core cognition, and (2) was importantly related to absences in our experience. To satisfy (1) and (2), however, perception is stronger than we need. All we require is that instances of zero are represented as absences as part of *cognition*. That zero is represented in cognition is supported by the data discussed above, but also Farennikova’s mismatch model can be adapted to *cognition* by holding that the relevant templates are projected on to experience (rather than the actual world). This move would block the inference to some interesting philosophical implications of our account (namely that there is an additional kind of absence perception at work in number cognition), but it would not impact our account of zero as satisfying the four constraints.

Moreover, an interesting point here is that if we *do* accept that multi-modal perception is possible, we would have discovered a *generality* to Farennikova’s model. Farennikova (2013) is primarily concerned with *visual* absences, but later conjectures that the account can be generalised to other modalities. The current proposal suggests that this is indeed possible, if one accepts that zero-cognition counts as a form of perception.

*Perception of instances of zero may not involve mismatches.* Interestingly, Farennikova holds that there may be challenges in applying her account to silence (Farennikova (2013), p. 452). She provides the following example; suppose I hear a lover’s footsteps grow silent as s/he is exiting a building. We have two cases, I could either (i) be mismatching an auditory template of his/her footsteps to surroundings, or (ii) I could be hearing silence simply by *failing* to hear his/her footsteps, because they are no longer audible. In (ii), however, I (supposedly) hear silence even though there is no mismatching occurring. Consider then the following structurally similar example. I am told to look at an array of three dots, and say every five seconds (given an auditory cue) how many objects are on the screen. Slowly (let’s say over a period of 2 mins) the dots are faded out, and at some point I presumably say that the number of dots on the screen is zero. We might argue that in that case I perceive an instance of zero simply by failing to perceive the dots, rather than by any sort of mismatch.

Our response is very quick; we reject the claim that failing to hear the footsteps or perceive the dots entails perception of an instance of silence or zero. Consider a case where I am told that the enemy soldiers will attack when the guns falls silent, or that I will win a million dollars if I can press a button within five seconds of there being zero dots on the screen. Suppose further that I am prone to daydreaming and lose concentration, staring up at the sky/ceiling. The enemy troops overrun my position, the million dollars slips through my fingers. A natural explanation in these cases is that I *didn’t hear* the silence, and I *didn’t see* zero dots on the screen. In both cases I fail to perceive the necessary stimulus, the silence of the guns in the first case and the dots in the second. It is thus at least plausible that failure to perceive a stimulus does not *entail* perception of an absence.

It is possible that despite this there might be cases where we do wish to say that an agent perceives an absence in virtue of a failure to perceive a stimulus rather than mismatching. My own intuition regarding Farennikova’s footsteps case is that it is a case of mismatching (unless of course the agent is not a particularly doting lover, and simply loses concentration and fails to mismatch, in which case I do not think they succeed in hearing the silence as in the cases above). However we can mobilise some empirical evidence in favour of the claim that there are cases of hearing silence involving mismatching. Hughes et al. (2001) played subjects series of tones at regular intervals, and then omitted a tone at random. They discovered that cortical activation was similar when the tone was omitted and when the tone was present. A natural interpretation of this data is that participants heard silence in these cases, and were doing so in virtue of a mismatch between the template induced by the regularity of the tones and the absence of a tone. It is thus plausible that absences of a similar kind to zero can be perceived in virtue of a mismatch, even if we have not conclusively argued that it must be so. We hope that further empirical data will help to inform this issue (for example by analysing cases of mismatch versus mere failure).

*Objecting to numerical cognition.* A different line of attack is to put pressure on the account of numbers as understood through numerical cognition. This has been pursued in the cognitive sciences by Núñez^38^ who argues that cultural factors are important in determining our epistemology of number (rather than it being “hardwired” by numerosity cognition and then integrated via bootstrapping procedure). In the philosophical literature, Buijsman (2017) argues that since numerical core cognition is available to infants, bootstrapping procedures fail to explain the ontogenetic delay in infants progressive understanding of the meanings of the numerals “1”, “2”, and “3”.

While we find the account of natural number as understood through bootstrapping procedures to be plausible, let us suppose that Núñez and Buijsman are in fact correct and that number epistemology should be understood through different means. Even accepting this, we contend that our account can still play an important role in the explanation of the epistemology of natural number.

Even if, as argued by Núñez, our arithmetical practices are substantially culturally grounded, it *remains* the case that such cultural practices containing a use for zero in their mathematics (in any reasonable sense of their possessing a similar concept to us) will use it in such a way that it can be used to represent an absence of positive numerosity. Therefore, while we may accept that our story of zero in this case is not *constitutive* of the concept of zero, it nonetheless has an important role to play in *explaining* our epistemology of zero. We would still use zero as a numerosity property to track absences of objects, absence perception and number cognition would still be features of our cognitive make-up, it is just that additional epistemological steps would need to be made in connecting the epistemology to the relevant cognition. Similar remarks apply to Buijsman (2017)’s account of the natural numbers as understood through Hume-style principles. Even if the epistemology is not *exactly* as we have described it, the underlying account of zero as a property of collections related to absence perception can be transferred to these cases.

Two objections relate to the cultural and infant specific ontogeny of zero. The first point is the following: Historically speaking, the use of zero was a comparatively late technological advance. Notation for zero first appears in around 400 BC in Babylonian mathematics, but it was not until the seventh century AD that we have record of it being used as a legitimate number in computation in the work of Brahmagupta.^39^

The historical and cultural story is mirrored by the ontogenetic development of children. As mentioned earlier, children of between 3 and 4 years of age can commonly count backwards to zero, however fail to integrate this adequately with their other numerical knowledge, often responding that one is smaller than zero.^40^

This raises the following objection: If zero is, as we’ve suggested, to be understood in terms of numerical absence perception, and this numerical absence perception is roughly hardwired in the human brain, then why is zero not a culturally and historically ubiquitous phenomenon, presenting contemporaneously with other numbers? Similarly in the case of child development, why is it, if zero is linked to the same perceptual faculties that give us knowledge of the very smallest cardinal numbers, that manifestation of its knowledge in the ordering of cardinal numbers presents much later (at around 6 years^41^).

We think that these criticisms can be answered. The key point is that just because something is a perceivable quality, does not necessarily mean that it is either easy or necessary to integrate into a theory of how we navigate the world. It might just be that in virtue of it being a kind of absence, zero is more difficult to integrate into conceptual systems. Indeed, we might hypothesise that the Mismatch Model would account for this; a failed search given a projection might well be more computationally and conceptually taxing than an immediate successful matching. This might then account for why children (and in fact mature adults) find it more difficult to compute with zero, and why a full understanding presents later.

Moreover, in the historical context it is unclear that we *need* to differentiate the perception of an absence of *some objects or stuff* from a the absence of *a positive cardinality* of that particular object or stuff. Suppose we are in some sort of ‘state of nature’ context where nutrition is scarce and key to survival. Is the distinction between whether I have an *absence of bananas* or *an absence of a positive numerosity of bananas* an important one? Roughly they come down to the same nutritionally relevant state of affairs; I have no bananas to eat.

Culturally speaking, the importance of distinguishing an absence of positive numerosity zero as opposed to absence *simpliciter* only becomes acute when we require relatively sophisticated ideas. Issues like the lending of money, positive and negative charge, and scientific theories utilising algebraic structures are the applications that require zero-concepts rather than mere absence. Zero then enters the picture in order to mediate the distinction between the positive and the negative, and provides an appropriate numerical property that can fill the required algebraic roles. But until such needs arise, there is no especial pressure to assert that a lack of numerical competence with a particular arithmetical concept is indicative of a lack of perception of the property corresponding to that concept.

Consider, as an analogy, the Pirahã people who have an approximate proto-arithmetic involving the words “one”, “two”, and “many”. In their proto-arithmetic one plus one is two, two plus one is many, and many plus one remains many.^42^ Does this threaten our epistemology of natural number as given by an integration of numerosity procedure with counting practices? It seems that the Pirahã have all the pieces (albeit a different counting procedure). Yet they fail to have a determinate concept for three, despite the fact that they are (presumably) regularly experiencing numerosity three perceptions.

We contend that this does not threaten the account of natural numbers as understood through numerosity perception and counting procedures. The reason is the same as in the zero case; while they may be having perceptions of a certain kind, this does not entail that they have sophisticated concepts that can apply to them. We do not need to maintain that counting and numerosity perception *has* to entail a satisfactory epistemology of number, just that it *can* for the particular arithmetical concepts we employ.^43^ Similarly for zero, it may very well be that one can have perceptions of instances of cardinality zero without possessing a concept and associated language to describe them.

## Conclusions and open questions

Before we identify some open questions, we recall the our Main Question for Realist philosophy from the Introduction:

**Main Question.** How should we should understand zero as a mathematical entity? In particular:

(A) How should we conceive of it ontologically?
(B) How is it able to represent nothingness?
(C) How is the cardinal number zero linked to similar uses of the term “0” in various technical scenarios?
(D) How can we provide an adequate epistemology for zero, in particular how we come to know about the cardinal number?

(A) we answered by arguing that zero is a collection-size property, just like the other numbers. For (B), we argued that it is able to represent nothingness by being instantiated by empty collections, and experienced through cognition/perception of absences. (C) was answered by noting that a commonality amongst several number systems involving a zero-like concept was some notion of absence in the zero-position, and that this then links to the role of zero-elements of algebraic structures. We responded to (D) by providing an account of concept acquisition via bootstrapping procedure (based on number cognition, counting procedures, an understanding of numerals linked to magnitudes), and an account of numbers as described via operations and algebraic roles within particular structures. As we’ve just argued, salient criticisms can be responded to.

There are, however, some open questions regarding zero both from a philosophical and cognition perspective. On the cognitive science side, whilst we have argued that zero can fulfil particular philosophical and mathematical functions if viewed as a kind of absence property, this part of the story has not yet been corroborated by empirical evidence, including neuronal activation. Part of the problem here is that it is unclear if there is *specific* neuronal activation corresponding to absence perception *in general*, or whether absence perception is rather a higher-level philosophical concept. Nonetheless, examining the similarities and differences between token perceptions of zero and other kinds of absence would be an interesting comparison in providing a full picture of how philosophical concepts and neurobiological facts interact. We therefore ask the following:

### Question 1

How does zero behave empirically with respect to other kinds of absence perception, including comparison of neuronal activation? For example, how does perception of zero compare with perception of silence from a neurobiological perspective?

Our next question concerns the fact that, as hinted to in the text, there seems no barrier to considering numerosities instantiated multi-modally. We know that human (and animal) perception of numerosity can be discriminated in different modalities, but this is not the same thing as analysing them in different modalities *simultaneously*. As far as we know, this has not been studied in detail, and so we ask the following for the specific case of zero:

### Question 2

What empirical data can we glean from studies involving the multi-modal instantiation of numerosity zero?

This also suggests a related question for the philosophical literature:

### Question 3

How does the possibility of perceiving a multi-modal absence inform our understanding of absence perception?

Finally, we leave open a question concerning how the account provided generalises to other cases. Earlier, we remarked that our account of zero can be integrated into other accounts of mathematical structures that make use of zero (e.g., the real numbers and set theory). However, we did not present detailed examination of those zero-concepts themselves. The following question is therefore important:

### Question 4

How should we understand zero-concepts in other areas of mathematics (e.g., the integers, real and complex analysis, and abstract algebra)?

We are hopeful that an answer to these questions will yield an account of mathematics on which the fundamental importance of zero as both a technical device and philosophically distinctive entity is developed. For now, we take ourselves to have made an initial step in this direction.

## Footnotes

1 For the purposes of this paper, we will use the term “cardinal number” to refer only to the finite cardinals.

2 We are sensitive to the fact that the term “Realism” is used in many and varied ways, and just make this assumption for the purposes of the current paper. See Jenkins (2008) (esp. Ch 1) for clarification of this issue.

3 They could, for example, be properties or concepts. Providing these are taken to be mind-independent, this would still constitute a variety of what Shapiro calls “object” realism (see Shapiro 2000, Ch. 2 (esp. §2) here), even though we might not think that properties or concepts are objects.

4 This is one of Benacerraf (1973)’s two motivating concerns on the nature of mathematical truth.

5 This attitude to the empty set (an object closely related to zero) is surprisingly widespread in the foundations of mathematics, especially amongst the early set theorists. Zermelo referred to it as *uneigentlich* (“improper”), Dedekind (Dedekind 1888, p. 797) excluded its consideration for reasons linked to problems of non-existence (and duly received criticism, along with Schröder in Frege 1917), and Fraenkel et al. (1973) regard its use as mere notational convenience. See Kanamori (2003) for further discussion.

6 Of course, not *every* apparent use of singular terms in mathematics is like this. Consider our use of the term “$\infty$” in real analysis (as in the expression“$\lim _{x \rightarrow \infty } (f(x) = \frac{2}{x^2 - 2x +2}) = 0$”). Here, we would not want to countenance $\infty$ as a genuine real number, despite the fact that it can have the surface features of a singular term. While there are some cases where this requirement is defeasible (say when we introduce the shorthand use of $\infty$ to talk concisely about limits), the case of zero has some disanalogies with this situation (not least because the introduction of $\infty$ as a real number with its required properties would result in contradiction, since (for example) the interval $[0, \infty )$ would have to be both bounded and unbounded, whereas zero can be regarded as a cardinal number without contradiction). Many thanks to Marcus Giaquinto for pressing me to be clear on this issue.

7 The first known instances are often taken to be the use of an empty space in calculation in China circa 500 BCE, and the existence of placeholders in notation in Babylonian mathematics in approximately 400 BCE. See Nieder (2016), p. 831.

8 There are, of course, distinctive technical roles for other numbers. “1”, for instance, denotes an identity element under multiplication. More generally, any cardinal number *n* will be unique in that it is the only number that numbers *n*-many things. We do not claim that the technical constraint only applies to zero, only that it is especially interesting in this context.

9 Views of this kind are Hellman (1989) and Shapiro (1997). It does not matter for current purposes whether the structuralism considered is *ante rem* or *in re*.

10 A second possibility, one that we will not consider in detail in the body of the text, is that if we think of cardinal numbers as structures of pure cardinality (i.e., the structure of the cardinal number 3 is the structure that contains three positions with no relations between them), then zero would be the empty structure. This might have some benefit for the Phenomenological Constraint, but it simply reduces the problem of epistemology of zero to the epistemology of the empty structure, and this seems like little philosophical gain. Since we consider a view very similar to this below, we will set it aside for now.

11 However, see Paseau (2009) for an interesting defence.

12 Depending on the object picked, we may have to tinker with certain axioms and then find models for pure set theories within those theories. Maddy (1990b) and Maddy (1990a) suggest taking the empty set to be an arbitrary doubleton $\{p, q \}$ (since she identifies objects with their singletons) and then to find a formal representation of $\mathbf {ZFC}$ in her system (which only contains impure sets). In this way, her position sits somewhere between realism and fictionalism (she herself refers to the empty set as a “fiction”); the empty set is an *object*, but significant *reinterpretation* is required to make pure set theory work. While we could immediately object at this point on the grounds of the Ontological Constraint (our theory of reference is being modified), since small modifications avoid this worry (e.g., just pick an arbitrary urelement *x*, and reject that $x = \{ x \}$) we set such issues aside.

13 These points are discussed further in Giaquinto (2017).

14 More tangentially, we might take zero as the beginning for the processes-as-objects account of $\aleph _0$ in Pantsar (2015), or as an initial point in the conceptual blending account provided in Núñez (2005).

15 See here Carey (2009), p. 67, and more generally Ch. 3.

16 See Dehaene (1997) for a book-length treatment of the matter, and Nieder and Dehaene (2009) for a relatively recent survey of the literature, and Dehaene and Brannon (2011) for a relatively recent collection. See Carey (2009), especially Chs. 4, 8 and 9 for a summary of the ontogenetic facts and an argument that the two systems are distinct.

17 See here Nieder and Dehaene (2009) for a review of some of the relevant literature here.

18 See also Dehaene (1997) for a presentation of some of the classic data on subitizing and the ANS in humans and animals. A clear and concise presentation of some of the data is also available in Carey (2009), Ch. 4. There is some controversy as to whether or not these two systems are actually both species of an underlying analog system, again see Carey (2009), Ch. 4 for discussion of this issue.

19 See here Bloom and Wynn (1997).

20 These descriptions can get very complex for especially large finite cardinals, as witnessed by Adam Elga and Augustín Rayo’s ‘Big Number Duel’. See Elga and Rayo (2007).

21 See here Mou and vanMarle (2014) and Wynn (1998).

22 An important caveat is in order here: though Mikulski exhibited genuine distance effects, Merritt et al. (2009) argue that Feinstein’s behaviour seemed to rather be linked to prior reward value of the non-zero array (i.e., She appeared to be confusing 1 and 0 more than 9 and 0 because 9 had a low reward-value in a comparison task whereas 1 had a high reward-value).

23 For a summary of the literature here, see Nieder (2016), pp. 838–841.

24 Merritt, Rugani, and Brannon are especially explicit here: “The studies reported here demonstrate the same type of conceptual understanding of zero. The rhesus monkey apparently treats the class of all empty sets as equivalent and appreciates that empty sets are smaller in numerical magnitude than nonempty sets. Most important, the current studies show that the monkey clearly appreciates empty sets as occupying a place on a numerical continuum.”(Merritt et al. (2009), p. 12.)

25 In Lipton and Spelke (2005).

26 Of course the predecessor function is not defined on 0, unless we are working with the integers (or a superstructure thereof). It is an interesting question, one that we leave as open, as to how this way of acquiring (partial) understanding of zero feeds into our epistemology of the integers.

27 In addition, children’s learning was accelerated, but order was not changed, by differences in educational background.

28 These questions are roughly paraphrased from the original example to suppress unnecessary details.

29 A separate issue, not considered in detail here, is whether we should regard this knowledge of zero as ultimately justified *analytically*, even if we obtain the concept from a kind of bootstrapping from multiple sources. A summary of the neo-Fregean position that justification of arithmetical knowledge is analytic is available in Tennant (2017), and a recent argument about concept acquisition and Neo-Fregeanism is available in Buijsman (2017).

30 See Sorensen (2008) and Farennikova (2013) for further discussion of this point. Good examples of theories of perception (in the case of sight) of this kind are those found (as Farennikova notes) in Marr (1982), Gibson (1966), and Dretske (1969).

31 See Sorensen (2008).

32 See Farennikova (2013).

33 We say relative, because it seems false that a token instance of silence must be accompanied by a *total* absence of sound waves. In music, for example, we can say that the performer(s) in John Cage’s 4’33” are silent, despite the fact that they may be making slight noises. The important point is that they are silent *relative* to the usual conventions of a musical performance. Some (e.g., Davies (2003), Ch. 1) treat this as indicative of the impossibility of silence, but see Sorensen (2008), p. 287 for an effective rebuttal.

34 This example is from Sorensen (2008), p. 269.

35 See, for example, Carey (2009) Ch 3. and p. 138 for a description and references.

36 See Meck and Church (1983) for the original study and Dehaene (1997) for discussion.

37 In paradigmatic humans; there is the difficult question of how to understand aurally-stimulated colour experience in synaesthetes.

38 See, for example, Núñez (2009) and Núñez (2011).

39 For an excellent survey of the history here, and an account of zero in the cognitive sciences, see Nieder (2016).

40 Again, see Nieder (2016) for a concise survey of some of the cognitive science literature.

41 See here Wellman and Miller (1986) for an early study into ontogenetic development of zero and Merritt et al. (2009), p. 3: “Together, research with babies and children suggests that both in symbolic and in nonsymbolic form, children’s concept of empty sets lags behind that of nonzero whole numbers. In addition, children’s understanding of zero does not happen all at once, but rather, children gradually acquire the important elemental properties of zero before those elements are fully integrated.”

42 See Gordon (2004) for the details.

43
Pantsar (2014) makes a related point (although he is less confident that the practice of the Pirahã can be described as proto-arithmetical) that the use of the perceptual approximate number system might underdetermine the eventual arithmetic adopted.
